# The Investigation of Flory–Huggins Interaction Parameters for Amorphous Solid Dispersion Across the Entire Temperature and Composition Range

**DOI:** 10.3390/pharmaceutics11080420

**Published:** 2019-08-19

**Authors:** Yiwei Tian, Kaijie Qian, Esther Jacobs, Esther Amstad, David S. Jones, Lorenzo Stella, Gavin P. Andrews

**Affiliations:** 1 Pharmaceutical Engineering Group, School of Pharmacy, Queen’s University Belfast, 97 Lisburn Road, Belfast BT9 7BL, UK; 2 Soft Materials Laboratory, Institute of Materials, École Polytechnique Fédérale de Lausanne (EPFL), CH-1015 Lausanne, Switzerland; 3 Atomistic Simulation Centre, School of Mathematics and Physics, Queen’s University Belfast, 7-9 College Park E, Belfast BT7 1PS, UK; 4 School of Chemistry and Chemical Engineering, Queen’s University Belfast, David Keir Building, Stranmillis Road, Belfast BT9 5AG, UK

**Keywords:** thermodynamic, amorphous solid dispersion, amorphous-amorphous phase separation, binary phase diagram, drug-polymer miscibility, Flory–Huggins interaction parameters

## Abstract

Amorphous solid dispersion (ASD) is one of the most promising enabling formulations featuring significant water solubility and bioavailability enhancements for biopharmaceutical classification system (BCS) class II and IV drugs. An accurate thermodynamic understanding of the ASD should be established for the ease of development of stable formulation with desired product performances. In this study, we report a first experimental approach combined with classic Flory–Huggins (F–H) modelling to understand the performances of ASD across the entire temperature and drug composition range. At low temperature and drug loading, water (moisture) was induced into the system to increase the mobility and accelerate the amorphous drug-amorphous polymer phase separation (AAPS). The binodal line indicating the boundary between one phase and AAPS of felodipine, PVPK15 and water ternary system was successfully measured, and the corresponding F–H interaction parameters (χ) for FD-PVPK15 binary system were derived. By combining dissolution/melting depression with AAPS approach, the relationship between temperature and drug loading with χ (Φ, T) for FD-PVPK15 system was modelled across the entire range as χ = 1.72 − 852/T + 5.17·Φ − 7.85·Φ^2^. This empirical equation can provide better understanding and prediction for the miscibility and stability of drug-polymer ASD at all conditions.

## 1. Introduction

Low solubility and bioavailability are significant issues for currently marketed drug products, as well as new chemical entities in pharmaceutical development. Many high potential small molecular candidates (~90%) belonging to the class II and IV in the biopharmaceutical class system (BCS) have very little chance to ever get to the market due to drug delivery difficulties [1,2]. The use of amorphous formulations, such as amorphous solid dispersion (ASD) and amorphous nanoparticles can significantly improve the drug dissolution and membrane permeability for oral dosage forms, hence improve the bioavailability and therapeutic windows for these potential drug candidates [3,4,5,6,7]. Extensive efforts have been made to assess the feasibilities of these enabling formulations and to maintain the product’s qualities and performances throughout its life cycle. These sound understanding can then be effectively built into the Quality by Design (QbD) framework and implemented for the development of process understanding and process control with predefined objectives. There has been a significant increase in the development of various predictive tools for ASD system where the propensity for phase separation in the mixture can be assessed [8,9,10]. More recently, the importance of amorphous-amorphous phase separation (AAPS) in ASD has also been highlighted in relation to the drug releases and physical stability of the formulation [11,12]. It is clear that further development of the informative framework is imperative, particularly for its role in establishing a useful strategy to pre-define the achievable quality attributes of ASD systems (performance and physical stability). 

Currently, two types of experimental methodologies are widely reported to determine the solubility limits of small molecule drug within the polymer matrix (phase diagram) [13]. One method is based on the detection of glass transition temperature after demixing of supersaturated ASD samples (high drug loadings) [14], the other is through measurement of the dissolution/melting of the crystalline drug within a polymer physic mixture [15,16]. However, due to the presence of high viscosity of the polymer in these testing samples, these two approaches provide overestimated or underestimated values on the solubility of the drug within the polymer [13,17]. Both the demixing method and the dissolution/melting method can only be used to physically measure the “equilibrium point” by using high drug loadings at high temperatures (e.g., higher than 70% *w*/*w* in the case of the felodipine and PVPK15 system). Further extrapolation on the phase diagram, based on high drug loading high temperature points, was suggested to be inappropriate, due to the potential changes on the interaction type or strength between drug and polymer at other drug loading/temperature conditions. Hiroshi et al. reported that, in the case of the naproxen and Eudragit^®^ E (EPO) system, the intermolecular interactions happen between the carboxylic acid group in naproxen and amine group of EPO at low drug loadings (20–70% *w/w*). Such intermolecular interactions switch to ester groups in the EPO when naproxen loading increases to 70% (*w*/*w*) or above [18]. Similar enhancements on drug-polymer interactions can also be found in many other cases where strong H-bonds was evidenced at certain drug compositions [19,20]. Karavas et al. reported that the strength of intermolecular interactions between felodipine and olyvinylpyrrolidone (PVP) was greatly enhanced when the drug loading reduces to 25% (*w*/*w*) or less [21]. Furthermore, temperature also acts as an important factor in the strength of drug-polymer intermolecular interaction. In the case of the ASD of octaacetylmaltose (acMAL) and celecoxib, the strength of the H-bonding between celecoxib and acMAL molecules reduced dramatically when the temperature of the ASD was raised to and above than the T_g_ of system. It was suggested that both the amount and the strength of the hydrogen bonding between acMAL and celecoxib were significantly reduced at high temperatures [22]. These changes in the strength and type of drug-polymer intermolecular interactions are extremely important for the establishment of the framework and predict the quality and performance of the final ASD dosage form.

**Figure 1:**
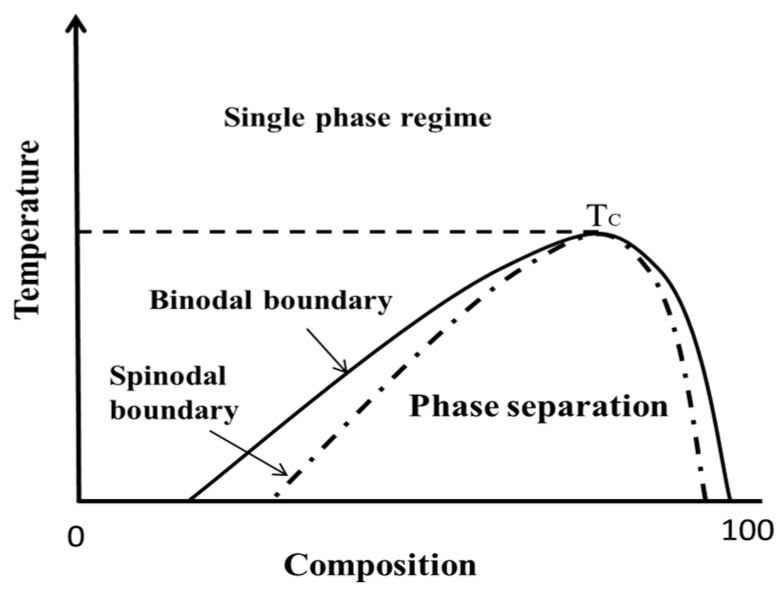
The schematic diagram for the binary system featuring upper critical solution temperature (UCST); the amorphous-amorphous phase separation (AAPS) occurs within both the binodal and spinodal boundary, with a nucleation barrier exists in the gap between binodal and spinodal.

In order to predict and assess the physical stability of the ASD formulation, the continuous monitoring of crystallization of drug from the amorphous system can be utilized. Many efforts were made to develop the crystallization kinetics of the amorphous drug in a different polymeric matrix [23,24,25,26]. The correlations between key physicochemical parameters of the polymers may be obtained and offer potential useful guidance for the selection of right carriers. However, it is vital to realize that such a method can be very time consuming, due to the viscosity of the ASD systems. It should also be highlighted that the crystallization of drug from an initial homogeneous ASD system may undergo various non-classical processes, such as amorphous-amorphous phase separation to lower its free energy prior to the nucleation and growth of crystalline from Reference [27,28,29,30]. In the case of ASD, when the AAPS occurs, a two-T_g_ system may be easily observed using conventional thermal analysis if the values of glass transitions for individual components are far apart. In fact, it has been widely reported that, for many ASD systems when moisture is induced, accelerated AAPS can be observed, e.g., felodipine with PVPVA, quinidine with PVP, quinidine with PVPVA, pimozide with PVPVA and pimozide with hydroxypropylmethylcellulose acetate succinate (HPMCAS) [31,32]. The potential of AAPS occurring in ASDs from a supersaturated state raises the critical point of binodal as the initial phase boundary between one phase and two-phase system [33,34]. From the demixing point of view, the experimental measurement of the AAPS boundary at various drug loadings/temperatures becomes significant for probing the stability of the initial homogenous ASD system [35]. When the ASD system sits outside the binodal curve, a typical one phase homogeneous glass solution may be expected, whilst, when the ASD system falls into the binodal curve, AAPS is expected to occur (Figure 1).

In light of this evidence, we have proposed to utilize this moisture-induced AAPS to probe the phase boundary for drug, polymer water ternary system. The binodal line from the ternary system indicating the boundary between one-phase and two-phase amorphous system has been directly measured, and the F–H interaction parameters χ for these critical points were successfully derived. The binary F–H interaction parameters exhibiting as χ_(drug-polymer),_ χ_(drug-water)_ and χ_(polymer-water)_ allow one to probe the level of intermolecular interactions (enthalpy and entropy of mixing) at the corresponded binodal boundary for the ternary system (compositions and temperatures). Therefore, χ_(drug-polymer)_ calculated from these conditions can be used to obtain the miscibility limits for drug-polymer ASD binary system. More importantly, due to the benefits of water mobility, this approach is capable of: (1) Measuring the phase separations of ASD samples at low temperatures and drug loadings; (2) reducing the impact of high viscosity provided by the polymer on the phase separation; (3) directly deriving the F–H interaction parameter χ_12_ for drug-polymer binary system at low temperatures; (4) combining with the χ_12_ obtained from dissolution/melting depression method to help the establishment of the relationship for the values of F–H interaction parameter with temperature and drug loading across entire range. This approach also allows one to construct the drug-polymer temperature-composition phase diagram with much-improved confidence.

## 2. Theory and Experimental Treatments

The process of phase transformation, and, subsequently, the formation of crystalline materials from amorphous-phase precursors are central to all fields’ science and process engineering. For example, in the context of crystal engineering from solution, the self-assembling of molecules during phase separation is fundamentally important for the formation of polymorphism, solvates, salt or cocrystals [36]. The thermodynamics underlying phase separation can be illustrated with generic phase diagrams featuring an upper critical solution temperature (UCST) in Figure 1. Fundamentally, as originally introduced by Gibbs and later developed by Cahn and Hilliard, the phase separation in the binodal regime is based on the fluctuations within a barrier to nucleation. The probability for nucleation increases with increasing supersaturation featuring a demixing and nucleation pattern [37]. In comparison to the binodal demixing nucleation mechanism, the spinodal decomposition region normally features with a bicontinuous pattern (no nucleation barrier). Commonly, either binodal demixing and spinodal decomposition happen very fast in high mobility solvent-solution cases. However, in the case of drug-polymer ASD with the involvements of the polymeric matrix, such phase separation process may be extremely slow, and the influences of various chemical structures of the polymer can also have significant impacts on the dynamics of this process [20]. 

In a drug-polymer ASD system, as discussed above, thermodynamic events, such as AAPS in solid dispersion cannot be directly observed at low drug composition or low temperature conditions, due to the high viscosity of the systems. Considering the amorphous drug and the polymer both being super-cooled liquid/glass at the experimental conditions (temperature), the crystallization of drug in a non-equilibrium ASD system may be preceded by the formation of a dense and disordered amorphous drug-rich phase (AAPS) [38,39]. To further speed up the AAPS, water (as a good plasticizer) can be used at controlled manner (dynamic vapor absorption) to increase the molecular mobility of the system significantly, in return, provides the opportunity to experimentally measure the AAPS at much lower temperatures and short timescales. Therefore, we have proposed to detect the AAPS boundary (the binodal line) of a drug-polymer system in the presence of moisture in this study. The ultimate goal of this experiment was to detect the boundary between one phase ASD and AAPS at the presence of water (uptake from high humidity environment) where the conditions favors the drug-polymer system at one phase. Passing these conditions, the intermolecular interactions formed within the drug-polymer binary system will be disrupted, and AAPS occurs. 

Flory–Huggins theory provides the simplest Gibbs free energy understanding of binary systems [40,41]. As a classical theory to describe the miscibility between two or more components, it is vital to recognize that the temperature and composition-dependence of F–H interaction parameter, *χ_ij_*, needs to be obtained across the entire range. The expansion of the equation on the ternary system can be described using Equation (1) [42,43]:

(1) $$\frac{\Delta G}{\mathrm{RT}}=n_{1}\ln\Phi_{1}+n_{2}\ln\Phi_{2}+n_{3}\ln\Phi_{3}+\left(\chi_{12}\Phi_{1}\Phi_{2}+\chi_{13}\Phi_{1}\Phi_{3}+\chi_{23}\Phi_{2}\Phi_{3}\right)\left(m_{1}n_{1}+m_{2}n_{2}+m_{3}n_{3}\right),$$

where *n_i_* is the number of moles of component i in a ternary system, $m_{i}=V_{i}/V_{3}$ is the ratio of the mole volume of the component i and of a reference component (water), *χ_ij_* (i<j) are the F–H interaction parameters between any two components of the mixture, Φ*_i_* is the volume fraction of component *i* in the ternary system. It should be noted that both the entropy of mixing and enthalpy of mixing should be treated as variables in the original F–H model, since strong interactions can lead to the formation of molecular complexes and specific volume changes. The molar volume for each component was considered as constant in this article and calculated based on its density at room temperature. In this case, we chose drug felodipine (FD), polymer PVPK15 and water three-component system that exhibits moisture-induced AAPS [31]. Number 1, 2, and 3 are representing for FD, PVPK15, and water, respectively. By building the model through experimental binodal points, we were able to solve the *χ*_12_ for the drug-polymer binary system at the corresponding conditions. 

The composition-dependent binary F–H interaction parameters were also utilized in ternary systems and demonstrated good agreements with the experimental data [44,45,46,47,48]. We applied the same approximation in this study where the concentration dependent of *χ*_12_ and *χ*_23_ were used to represent the interactions parameters of FD-PVPK15 and water-PVPK15, respectively. These two χ_12_ and χ_23_ were also used to modelling the FD-PVPK15-water ternary systems described as [46,47]:

(2) $$\chi_{12}\left(u_{1}\right)\left(u_{1}=\frac{\Phi_{1}}{\Phi_{1}+\Phi_{2}}\right),$$

(3) $$\chi_{23}\left(u_{2}\right)\left(u_{2}=\frac{\Phi_{2}}{\Phi_{2}+\Phi_{3}}\right).$$

As we are introducing the composition-dependence of χ_12_ and χ_23_ for modelling of FD-PVPK15-water ternary system, it is critically important to realize that both χ_12_ and χ_23_ are functions of $u_{1}$ and $u_{2}$ Equations (2) and (3) whose values are based on the volume ratio of $\Phi_{1}$ and $\Phi_{2}$ in binary systems of FD-PVPK15 and water-PVPK15 respectively. Thus, by removing the water out of FD-PVPK15-water system, the composition-dependence of χ_12_ ($u_{1}$) for FD-PVPK15 binary system will not be altered. In this study, the F–H interaction parameter for amorphous FD-water, χ_13_, was considered as a drug loading independent parameter, due to the hydrophobicity of FD. Very limited water (0.02% *w*/*w* at various temperatures) was absorbed into the pure amorphous FD throughout all conditions of our dynamic vapor absorption experiments. Although the interaction between drug and polymer may be completely disrupted by the induced water with the advent of phase separation, there is no evidence suggesting that the interactions between FD and PVPK15 within the one phase ASD were disrupted prior to the AAPS [49]. Therefore, we have proposed in this study to identify the conditions for binodal boundary (critical position between one phase and AAPS), where the interaction parameter χ_12_ for FD-PVPK15 should be captured at corresponding conditions Equation (1). With this approach, the χ_12_ between drug and polymer obtaining from a ternary system at binodal boundary may also be used for the understanding of FD-PVPK15 binary system.

**Figure 2:**
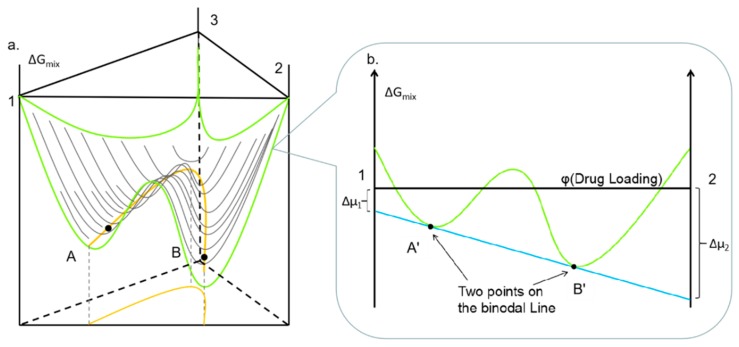
(**a**) Schematic diagram of Gibbs free energy surface (green line) of a ternary system at a constant temperature; the orange line representing the binodal curve (Figure 1) at constant temperature; (**b**) Illustration of Gibbs free energy profile for a binary system at a constant temperature using common tangent rule (blue line).

Figure 2 illustrates the theory of obtaining binodal points in a ternary system at various drug loading and temperature conditions. Figure 2b is a transverse section of Figure 2a; and it illustrated the Gibbs free energy profile for a binary system at a constant temperature. At the binodal point in a binary system, the chemical potential of component 1 and 2 at point A’ are equal to the point B’, which can be described by:

(4) $$\{\begin{array}{c} \Delta \mu_{1}\left(\Phi_{1}^{A{}^{\prime }},\Phi_{2}^{A{}^{\prime }}\right)=\Delta \mu_{1}\left(\Phi_{1}^{B{}^{\prime }},\Phi_{2}^{B{}^{\prime }}\right)\\ \Delta \mu_{2}\left(\Phi_{1}^{A{}^{\prime }},\Phi_{2}^{A{}^{\prime }}\right)=\Delta \mu_{2}\left(\Phi_{1}^{B{}^{\prime }},\Phi_{2}^{B{}^{\prime }}\right) \end{array},$$

where Δ*μ_i_* is the changing of the chemical potential from initial to equilibrium state. Thus, in the binary system, the Δ*μ_drug_* and Δ*μ_polymer_* at drug loading A’ are equal to point B’. 

They are two points on the binodal line of the binary system. A similar rule applies to the ternary system where the binodal line is illustrated by the orange line in Figure 2a. Similar to the binary system, there are two points (A and B) on the binodal line in the ternary system where the chemical potential of three components at point A are equal to point B, which is given by:

(5) $$\{\begin{array}{c} \Delta \mu_{1}\left(\Phi_{1}^{A},\Phi_{2}^{A},\Phi_{3}^{A}\right)=\Delta \mu_{1}\left(\Phi_{1}^{B},\Phi_{2}^{B},\Phi_{3}^{B}\right)\\ \Delta \mu_{2}\left(\Phi_{1}^{A},\Phi_{2}^{A},\Phi_{3}^{A}\right)=\Delta \mu_{2}\left(\Phi_{1}^{B},\Phi_{2}^{B},\Phi_{3}^{B}\right)\\ \Delta \mu_{3}\left(\Phi_{1}^{A},\Phi_{2}^{A},\Phi_{3}^{A}\right)=\Delta \mu_{3}\left(\Phi_{1}^{B},\Phi_{2}^{B},\Phi_{3}^{B}\right) \end{array}.$$

To directly solve the binodal line for a ternary system, a partial derivative functions for the chemical potential of the individual component may be used [42]:

(6) $$\{\begin{array}{c} \frac{\frac{\partial \Delta G}{\mathrm{RT}}}{\partial n_{1}}=\frac{{\Delta \mu }_{1}}{\mathrm{RT}}=\ln\Phi_{1}+\left(1-\frac{m_{1}}{m_{2}}\right)\Phi_{2}+\left(1-m_{1}\right)\Phi_{3}+m_{1}[\zeta_{1}\left(\Phi_{2}+\Phi_{3}{)}^{2}+\zeta_{2}\Phi_{2}{}^{2}+\zeta_{3}\Phi_{3}{}^{2}\right]\\ \frac{\frac{\partial \Delta G}{\mathrm{RT}}}{\partial n_{2}}=\frac{{\Delta \mu }_{2}}{\mathrm{RT}}=\ln\Phi_{2}+\left(1-\frac{m_{2}}{m_{1}}\right)\Phi_{1}+\left(1-m_{2}\right)\Phi_{3}+m_{2}[\zeta_{2}\left(\Phi_{1}+\Phi_{3}{)}^{2}+\zeta_{1}\Phi_{1}{}^{2}+\zeta_{3}\Phi_{3}{}^{2}\right]\\ \frac{\frac{\partial \Delta G}{\mathrm{RT}}}{\partial n_{3}}=\frac{{\Delta \mu }_{3}}{\mathrm{RT}}=\ln\Phi_{3}+\left(1-\frac{1}{m_{1}}\right)\Phi_{1}+\left(1-\frac{1}{m_{2}}\right)\Phi_{2}+\zeta_{3}{\left(\Phi_{1}+\Phi_{3}\right)}^{2}+\zeta_{1}\Phi_{1}{}^{2}+\zeta_{2}\Phi_{2}{}^{2} \end{array},$$

where $\zeta_{i}$ was defined as:

(7) $$\{\begin{array}{c} \zeta_{1}=0.5\;\times \;(\chi_{12}+\chi_{13}-\chi_{23})\\ \zeta_{2}=0.5\;\times \;(\chi_{12}+\chi_{23}-\chi_{13})\\ \zeta_{3}=0.5\;\times \;(\chi_{13}+\chi_{23}-\chi_{12}) \end{array}.$$

The binodal line that gives the boundary between one phase and AAPS can be found by solving Equation (5) along with the definitions of the chemical potentials in Equations (6) and (7). In this way, the points *A* and *B* defined in Equation (5) will lie at the two extremes of a tie line.

Instead of solving Equation (5) directly, we have found a numerically more convenient way to deal with an equivalent variation problem. In particular, we define a dimensionless objective function as [50]:

(8) $$F_{\mathrm{OBJ}}\left(\chi_{12},{\;\Phi }_{1}^{B},\Phi_{2}^{B},\Phi_{3}^{B}\right)=\sum_{i=1}^{3}{\left[\Delta \mu_{i}\left(\Phi_{1}^{A},\Phi_{2}^{A},\Phi_{3}^{A}\right)-\Delta \mu_{i}\left(\Phi_{1}^{B},\Phi_{2}^{B},\Phi_{3}^{B}\right)\right]}^{2}.$$

The objective function is exactly zero when Equation (5) holds and larger than zero elsewhere. In practice, we start from the experimental values of $\Phi_{1}^{A},\Phi_{2}^{A},\Phi_{3}^{A}$, along with $\chi_{13}$, $\chi_{23}$, and all the molar volumes, and minimize $F_{OBJ}\left(\chi_{12},\;\Phi_{1}^{B},\Phi_{2}^{B},\Phi_{3}^{B}\right)$ in order to find the value of $\chi_{12}$ and $\Phi_{1}^{B},\Phi_{2}^{B},\Phi_{3}^{B}$ which satisfy Equation (5). To avoid the trivial solution, $\Phi_{i}^{A}=\Phi_{i}^{B}$, we use a constrained optimization algorithm which insures that $\overset{3}{\underset{i=1}{\sum }}{\left(\Phi_{i}^{A}-\Phi_{i}^{B}\right)}^{2}$ does not vanish. To rule out spurious local minima of the objective function, the minimal value of $F_{OBJ}$ is monitored so that it is numerically very close to zero. Note that this minimization procedure is in principle exact and the uncertainty in the solution is a consequence of the uncertainty in the experiment. The MATLAB^®^ scripts used to minimize the objective function, Equation (8), are included in Supplementary Materials A. To completely solve the Equations (6), (7), (8), the temperature -dependent interaction parameters for drug-water (χ_13_) and temperature and drug loading dependent interaction parameters for PVPK15-water (χ_23_) were fitted using separated equations (Supplementary Materials B).

The drug loading dependent interaction parameter between FD-PVPK15, $\chi_{12}(u_{1},\;T$), obtained at different temperatures by the AAPS approach may be further used to obtain a better understanding of the FD-PVP15 in relationship with both temperatures and drug loadings:

(9) $$\chi_{12}\left(u_{1},T\right)=A+\frac{B}{T}+C\times u_{1}+D\times u_{1}{}^{2},\;$$

where A, B, C, and D were defined by the values of F–H interaction parameter χ_12_ from binodal points of FD-PVPK15-water ternary phase diagram. In the FD and PVPK15 binary system, the F–H interaction parameter for drug and polymer, $\chi_{12}(u_{1},\;T$), can be simplified as $\chi_{12}(\Phi_{1},\;T$) in the binary system described as:

(10) $$\chi_{12}\left(\Phi_{1},T\right)=A+\frac{B}{T}+C\times \Phi_{1}+D\times \Phi_{1}{}^{2}.$$

Very recently, both temperature and drug loading dependence of F–H interaction parameter have been investigated using Equation (10) for the first time to understand the miscibility for amorphous solid dispersions [51]. However, the fitting constants of A, B, C and D for χ_12_ were obtained based on the systems annealed at high temperature conditions. It should be highlighted that the temperature and drug loading dependence of F–H interaction parameter can be useful if only the relationship is identified based on a wide range of temperatures and drug loadings. Our approach directly addresses this issue in the literature, allows one to directly derive the χ_12_ at low temperatures and drug loadings. These values of χ_12_ can then be combined with the values obtained using dissolution/melting depressions methods at high temperatures and drug loadings to fit the Equation (10), where the fitting constants A, B, C and D may be used to illustrate the temperature and drug loading dependence of F–H interaction parameter across the entire conditions. 

With the A, B, C and D four fitting constants obtained across the entire temperature and drug loading range, the final drug solubility curve for FD-PVPK15 binary system may be extrapolated with improved confidence, Equation (11) has been used [15]:

(11) $$\left(\frac{1}{T_{m}}-\frac{1}{T_{m}^{0}}\right)=-\frac{R}{\Delta H_{m}}\left[\ln\Phi_{1}+\left(1-\frac{1}{m}\right)\Phi_{2}+\chi_{12}\Phi_{2}^{2}\right],$$

where T_m_^0^ is the melting point of the pure drug, T_m_ is the melting point of the drug-polymer mixture, ΔH_m_ is the heat of fusion of the drug, *m* is defined as *N_B_/N_A_*, *N_A_* and *N_B_* is the molecular volume of drug and polymer respectively, χ_12_ is obtained from the Equation (10) at varying temperatures and drug loadings. Through the combination of the mathematic modelling and experimental measurements, we were able to directly derive all the values of F–H interaction parameter, χ, for the drug, polymer binary system as the functions of temperature and composition. The drug-polymer interaction parameter $\chi_{12}(\Phi_{1},\;T$) was further used to model the binary phase diagram for FD and PVPK15 at the corresponding conditions. 

## 3. Materials and Methods

### 3.1. Materials

Felodipine (FD, 384.25 g/mol), was generously gifted by AstraZeneca (Maccsfield, UK)), poly(vinylpyrrolidone) (PVPK15, molecular weight from 7000 to 11,000 g/mole) was a generous gift from Ashland (Kidderminster, UK). The purified water was obtained using PKPD Millipore water purification system 7, the resistivity of water was 18.2 MΩ·cm (MERCK, Millipore, Watford, UK).

### 3.2. Methods

#### 3.2.1. Sample Preparation

The physical mixture of FD and PVPK15 with drug loading from 0% to 100% (at 10% intervals) was prepared with a ball mill mixer (Retsch MM200, Haan, Germany). The drug and polymer mixture (totaling 500 mg) were placed into a 25 mL milling chamber with two stainless steel balls and oscillated at 20 Hz for 8–10 min.

Pure amorphous FD and ASD samples (5–10 mg) with different drug loadings (35%, 40%, 45%, and 50%) were prepared via heat-cool cycle using differential scanning calorimetry (DSC8000, Perkin Elmer, Beaconsfield, UK) at a heating rate of 20 °C/min from 20 to 150 °C held for five min, then it was cooled to −40 °Cat the rate of 100 °C/min (5–10 mg, aluminum pan without lid). After the initial heat and cool cycle, the sample was heated again to 150 °C with the rate of 100 °C/min to confirm the amorphization of the drug and uniformity of the ASD. The freshly prepared ASD samples (within the DSC pan) were then directly placed within the DVS for moisture-induced AAPS studies.

#### 3.2.2. Fourier Transform Infrared Spectroscopy (FT-IR)

Intra/intermolecular interaction between drug and polymer within ASD at various drug weight fractions (0%, 30%, 40%, 45%, 50%, 60%, 70%, 80%, 100%) were characterized by the FT-IR. All samples were scanned from 4000 cm^−1^ to 600 cm^−1^, with 2.0 cm^−1^ resolution and 64 scans per spectrum.

#### 3.2.3. Dynamic Vapor Absorption (DVS)

Prepared amorphous samples were annealed in the DVS for various times with constant water pressure (90% relative humidity, RH). Six hours were selected for all the ASD samples where most of the systems have reached steady state after five hours at 90% RH (steady state, Supplementary Materials C). Compared with a moisture-induced system, Luebbert et al. suggested that more water molecules are absorbed into the sample when the AAPS occurs, whilst less moisture will be absorbed by the sample when drug crystallization occurs [52]. This suggests that the equilibrium can be reached for ASD when a constant mass was obtained during high humidity AAPS state. In our experiment, the steady state was reached before six hours at 90% RH for all samples. After the annealing, the water pressure was then reduced to 0% to allow the samples to dry within DVS until a constant mass was obtained (up to two days at 0% RH, 20 °C). After this drying procedure, the ASD was subjected to thermal analysis and micro-Raman chemical mapping. The final water content for samples with confirmation of AAPS was also detected by the DVS. The additional weight of the samples was recorded as the composition of water. Once confirmed the conditions for AAPS, all the samples were repeated at least three times.

DVS was also used to detect water vapor absorptions for individual components, felodipine and PVPK15. The moisture uptake for these systems can be used to calculate the interaction parameters χ_13_ (aFD-water) and χ_23_ (PVPK15-water) Equation (B1) (Supplementary Materials B). In a typical procedure, approximately 50 mg of amorphous FD and PVP K15 were individually annealed in the DVS sample holder (mesh) at 30 °C, 40 °C, 50 °C, and 60 °C The pure amorphous FD was prepared by DSC using a previously described method (Section 3.2.1). The relative humidity was increased from 5% to 90% at 10% increment. In the DVS method, the delta mass over delta time (dM/dt) at each humidity step was chosen to be a minimum of five min, and maximum of 300 min equilibrium time with 99% equilibrium ratio. The short initial equilibrium time was set to avoid the crystallization of amorphous FD during DVS experiment, whilst long equilibrium time was to accommodate the absorption of water in PVPK15 samples. Due to the lower tendency of aFD to crystallization in comparison to many other pure amorphous drugs, we were able to obtain steady weight increments at all experimental conditions. The data of absorbed water mass as a function of humidity level was used to calculate the χ_13_ and χ_23_ using Equations (B1) and (B2) (Supplementary Materials B), the values of χ_13_ and χ_23_ were further extrapolated to the temperature and drug loading range for modelling of AAPS in the ternary system using Equations (B1)–(B4) (Supplementary Materials B).

#### 3.2.4. Thermal Analysis

For the verification of AAPS in FD, PVPK15 and water ternary system, thermal analysis was used. The binodal line may be used as the boundary between one phase (single T_g_) and AAPS (two T_g_s). The identification of the AAPS was achieved by the High-Performance Differential Scanning Calorimetry (DSC8000, PerkinElmer, Beaconsfield, UK) at heating rate of 200 °C/min until 180 °C. All samples that showed one T_g_ after annealing procedure were classified as one phase, which indicated that the conditions (temperature and drug loading) were still above the binodal curve of the ternary system. Changes on the DVS annealing conditions, e.g., lower the temperature, may result in the appearance of AAPS.

The ball mill mixtures with drug loading from 0% to 100% (at 10% intervals) of FD and PVPK15 binary systems were also prepared as ASDs using the DSC, the T_g_ of freshly prepared ASD was recorded at the middle of the heat capacity change. All the experiments were repeated at least three times. The predicted T_g_ was also calculated using the Gordon–Taylor equation:

(12) $$T_{g,\mathrm{mix}}=\frac{w_{1}T_{g,1}+{\mathrm{kw}}_{2}+T_{g,2}}{w_{1}+{\mathrm{kw}}_{2}}$$

where T_g,mix_, T_g1_, T_g2_ are glass transition temperatures of the ASD, pure component FD and PVPK15 respectively, w_i_ is the component weight ratio, k is defined as *ρ*_1_T_g1_/*ρ*_2_T_g2_, here *ρ* are the density of pure component [53].

#### 3.2.5. Raman Chemical Mapping

The Raman chemical mappings of the freshly prepared ASD samples and annealed samples were performed by a Raman Microscope 300 (PerkinElmer, Beaconsfield, UK. Using a 20x objective lens, the diameter of the Raman spot was yielded at 10 µm, and the space of 3µm was used for surface mapping. All samples were recorded in the Raman shift range from 1750 to 1300 cm^−1^ were selected at the main characteristic regions. The spectra were analyzed by the Grams/AI version 7.02 from Thermo GalacticTM (Thermo Fisher Scientific, Waltham, MA USA). All the maps were compared with the spectrum of the amorphous FD (aFD) collected at the same parameters.

#### 3.2.6. Statistical Analysis

The statistical analysis for effects of temperature and water activity on F–H interaction parameters at system aFD-water and aPVPK15-water was carried out using Kruskal-Wallis one-way ANOVA followed by Tukey-Kramer post hoc tests; *p <* 0.05 was considered as significant.

## 4. Results and Discussions

### 4.1. Variations of Drug-polymer Interaction at Different Drug Loading

**Figure 3:**
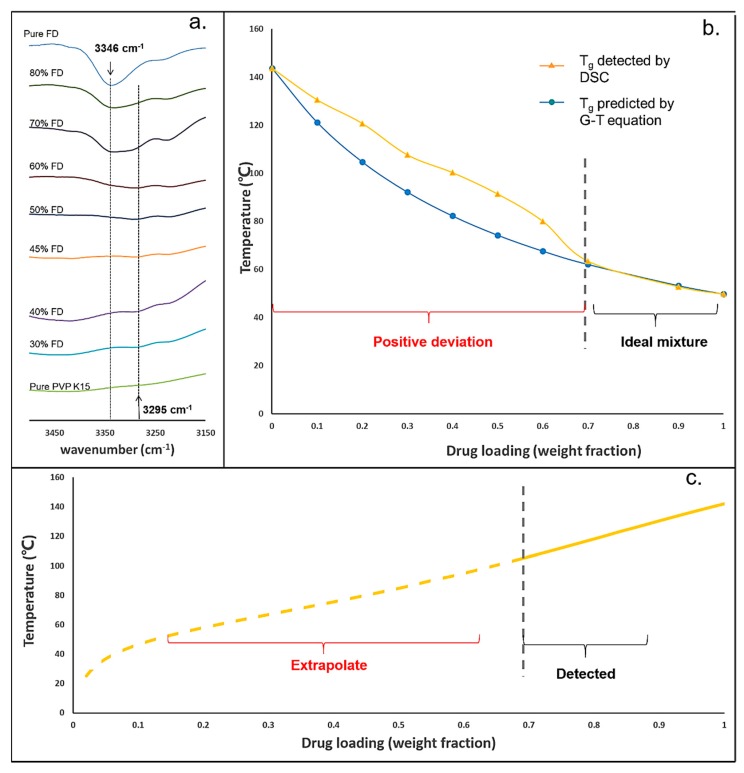
(**a**) The selected Infrared spectra of aFD-PVPK15 containing FD from range 0% to 100% (from top to bottom); (**b**) The yellow line represents the values of T_g_ of aFD and PVPK15 ASDs measured by DSC, the blue line represents the T_g_s predicted by the Gordon-Taylor equation (ideal solution) (*n* = 3); (**c**) The solubility line of system aFD and PVPK15 predicted using dissolution/melting depression data [54].

The IR spectra of FD-PVPK15 ASDs containing different weight fractions of FD were shown in Figure 3a. A clear indication of the drug-polymer interaction can be observed at the –NH stretch region (FD), where the vibration peak position was shifted from.

Wavenumber 3346 cm^−1^ for high drug loadings to 3295 cm^−1^ for low drug loadings. The height of peak located at 3346 cm^−1^ and 3295cm^−1^ were used to indicate the strength of hydrogen bonding between FD and PVP K15 within the ASDs. The height of the vibration peak at 3346 cm^−1^ was also reduced dramatically when the FD content reduces to 70–60% *w*/*w*, and replaced by a clear vibration peak at 3295 cm^−1^. Lower position for –NH stretch (large shifted in comparison to the pure FD) normally suggests a stronger intermolecular hydrogen bonding, as previously reported in the literature [31].

By using FT-IR, it was demonstrated that the intermolecular hydrogen bonding between –NH groups of FD and C=O groups of PVPK15 became much stronger when the drug loading was lower than 70 % *w*/*w* in the ASDs. Whilst, when the drug loading was higher than 70% *w*/*w*, additional drug-drug interactions were presented. Similar suggestions on the changes of FD-PVPK15 intermolecular interaction may be obtained through thermal analysis method, where the experimental values of T_g_ for ASDs can be compared to the theoretical values predicted by Gordon–Taylor (G–T) equation. The results were shown in Figure 3b. The yellow line represents the T_g_ values of aFD-PVPK15 ASDs measured by DSC, and the blue line represents the predicted values using the Gordon-Taylor model (based on the ideal solution). The standard deviation of detected T_g_ values were less than 2%, and the error bars were covered by the symbols. Positive derivations on the experimental T_g_ values of aFD-PVPK15 ASDs can be observed when FD contents were lower than 70% *w*/*w*, which suggested a stronger drug-polymer intermolecular interaction and reduced free volumes in these ASDs [55]. Crowley et al. also suggested that the deviation of glass transition temperature was attributed to the changes of homonuclear (FD-FD, PVPK15-PVPK15) and heteronuclear (FD-PVPK15) interaction [44]. In the case of positive deviation, the homonuclear interactions were weaker than heteronuclear interactions suggesting the two components like each other more than themselves at these drug compositions. Therefore, when the FD loading was lowered than 70% *w*/*w*, FD-PVPK15 intermolecular interactions were dominated in the ASDs. Through the combination of both FT-IR and DSC, it was confirmed that the strength of FD-PVPK15 intermolecular interaction within the ASDs was significantly enhanced at FD loadings of 70% *w*/*w* or less.

### 4.2. The Issues Associated with the Prediction of Drug Solubility Limits in Polymer

Most drugs and pharmaceutical polymers are solid or highly viscous at ambient conditions, measuring the drug solubility within a solid polymer under these conditions is not feasible [15]. As a consequence, predictions from the reported methods are associated with a degree of uncertainty. For example, several approaches were reported to obtain the solubility limits for the drug in the polymeric matrix, where high temperatures were used to accelerate the equilibration of the system [15,16]. The measured solubility points from high temperatures were then extrapolated to low temperatures to obtain the solubility limits with constant or temperature-dependent F–H interaction parameters. An example of the FD solubility curve in PVPK15 constructed using dissolution/melting method with temperature-dependent F–H interaction parameters was shown in Figure 3c. The solubility points at drug loadings higher than 70% *w*/*w* (solid line) were experimentally measured using thermal analysis (at T > 120°C), subsequently, the solubility points at lower temperatures (T < 120 °C) were extrapolated using the relationship obtained at high temperatures. However, as we already discussed that the level of FD-PVPK15 intermolecular interaction had significantly changed when the FD content was lower than 70% *w*/*w*. Therefore, the enhanced FD-PVPK15 intermolecular interactions were not captured in the high temperature dissolution/melting experiments (ideal mixing); hence, an underestimation on the extrapolated solubility value was expected [13].

### 4.3. Measurements for χ_13_ and χ_23_ Using DVS

**Figure 4:**
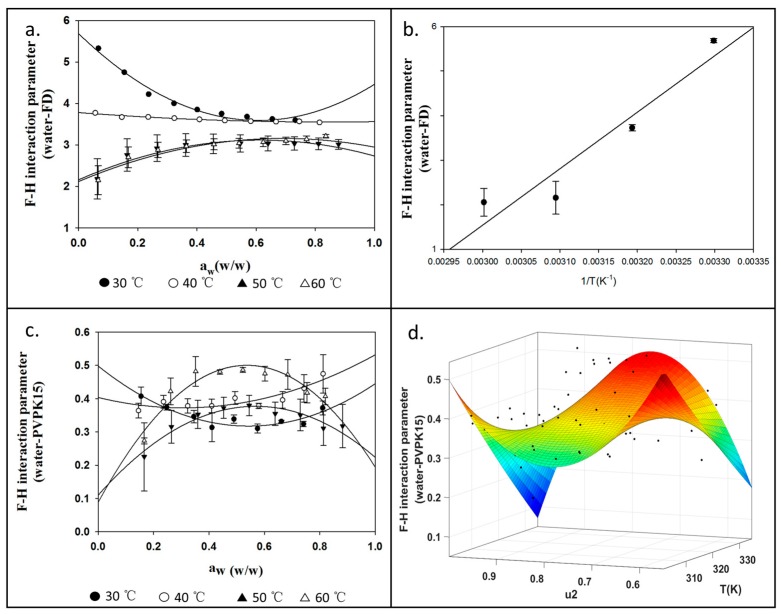
(**a**) Calculated F–H interaction parameter χ_13_ for aFD-water system at 30 °C, 40 °C, 50 °C and 60 °C; (**b**) The interaction parameter of χ_13_ (aFD-water) as a function of temperature by fitting Equation (B3) (Supplementary Materials B) (χ = A + B/T); (**c**) Calculated F–H interaction parameter χ_23_ for PVPK15-water at 30 °C, 40 °C, 50 °C and 60 °C (**d**) The interaction parameter of χ_23_ as functions of temperature and drug loading (u_2_) by fitting Equation (B4) (Supplementary Materials B).

In order to implement our concept of measuring the AAPS boundary in the aFD, PVPK15 and water ternary system, we need to solve the Equations (6)–(8). The unknown parameters preventing us to further solve these equations are the temperature-dependent F–H interaction parameters χ_13_ (aFD-water) and temperature and drug loading dependent χ_23_ (PVP K15-water). Separate experiments were conducted using DVS for aFD-water and PVPK15-water systems. The water activities in aFD and PVPK15 were reflected in the number of water uptakes at various humidity and temperature conditions. Using Equations (B1) and (B2) (Supplementary Materials B), the χ_13_ and χ_23_ were calculated (Supplementary Materials B). The value of χ_(aw)_ in the Equation (B1) (Supplementary Materials B) between aFD-water and PVP K15-water under different water activities and temperature 30 °C, 40 °C, 50 °C and 60 °C were shown in Figure 4a,c respectively. Values χ(0) in the Equation (B2) (Supplementary Materials B) were considered as the static drug loading independent F–H interaction parameter for drug-water (χ_13_) system at different temperatures [56]. Data of χ_13_ derived at various temperatures were then fitted by Equation (B2) (Supplementary Materials B), and the curve of χ_13_ as a function of temperature, as illustrated in Figure 4b. The values of the χ_23_ (PVPK15-water) measured by the DVS data and Equation (B1) (Supplementary Materials B) were then fitted by both drug loading and temperature-related function Equation (B4) (Supplementary Materials B). Figure 4d illustrated the fitted surface of Equation (B4) (Supplementary Materials B), which indicated the relationship between temperature, drug loading and PVPK15-water interaction parameter, χ_23_. Black points indicated the χ_23_ data derived by the Equation (B1) (Supplementary Materials B) from the experiment.

The summary of χ_13_ (aFD-water) and χ_23_ (PVPK15-water) were shown in Table 1, the values of χ_13_ were generally larger than χ_23_, and both of them decrease as the temperature increase. In the case of amorphous FD and water, A and B were fitted as −36.51 and 12684 with the goodness of fit (R^2^) yielded of 0.91. The interaction parameters between PVPK15 and water were fitted with Equation (B4) (Supplementary Materials B) and yielded coefficients were shown in Table 1. It is clear to see that with PVPK15 being more hydrophilic than amorphous drug FD with low F–H interaction parameter values (close to zero or negative).

### 4.4. Detection of AAPS in aFD-PVPK15-Water Ternary System

**Figure 5:**
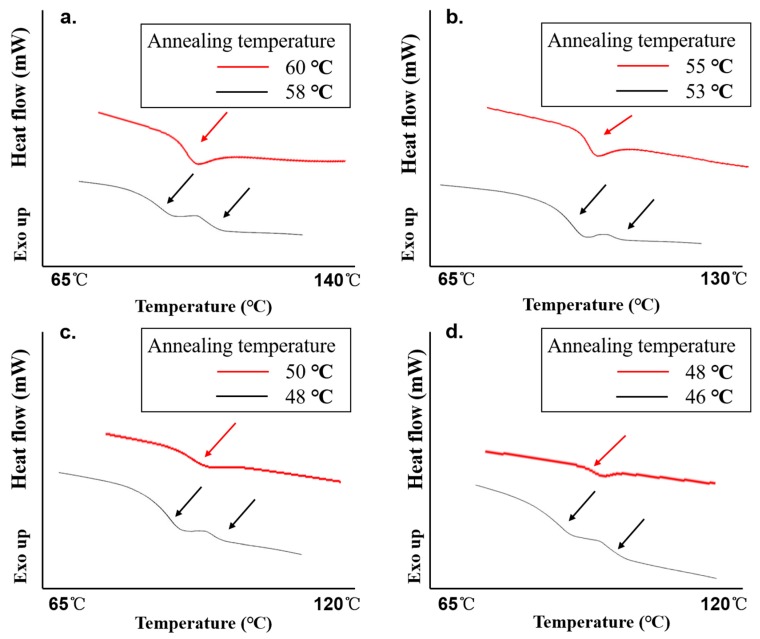
The thermograms of FD-PVPK15 ASDs at drug loading. (**a**) 35% drug loading; (**b**) 40% drug loading; (**c**) 45% drug loading; (**d**) 50% drug loading; the red lines represent the ASDs after annealed at the conditions above the binodal curve, and black lines represent the dried ASDs after annealed at conditions below the binodal curve (Table 2).

Because the type and strength of the drug-polymer interactions are different at various temperatures and drug loadings for FD-PVPK15 ASDs, the extrapolation of the interaction parameters obtained from high temperatures are not reliable for low temperatures. With the presence of water, we were able to introduce the AAPS in FD-PVPK15 ASD at much lower temperature conditions where the phase separations at drug loading less than 70% *w*/*w* were directly detected. The AAPS was firstly confirmed by DSC, where single T_g_ was obtained for systems annealed at higher temperature, whilst for systems annealed at a lower temperature, two glass transitions were obtained. If the ASDs were annealed within the binodal conditions for prolonged periods, the crystallization would occur from the amorphous drug-rich domains (Supplementary Materials D, Figure S2). The appearance of two-T_g_s system from DSC was first used to verify the conditions (temperature) for the boundary between one phase and AAPS. The represented examples of DSC results were shown in Figure 5. The red lines were the thermograms of the FD-PVPK15 ASD samples (with drug loadings from 35% to 50% *w*/*w*) showing one Tg after annealing (outside the AAPS). Black lines were the thermograms of the samples showing two T_g_s with varying drug loadings, which indicated the annealing conditions for these samples were inside the AAPS. Through this approach, the binodal curve (critical positions between one phase and AAPS) for FD-PVPK15-water ternary system was defined at temperatures 59 °C, 54 °C, 49 °C and 47 °C containing FD of 35%, 40%, 45% and 50% *w*/*w* respectively (Table 2). Recently, a very detailed illustration for the formation of AAPS in binary drug-polymer ASD has been reported where the drug-rich and polymer-rich domains at various time scales can be modelled [57]. In conclusion, two T_g_ systems were shown in black lines in Figure 5 when a suitable annealing temperature was selected, whilst one T_g_ systems can be observed at higher annealing temperature.

**Figure 6:**
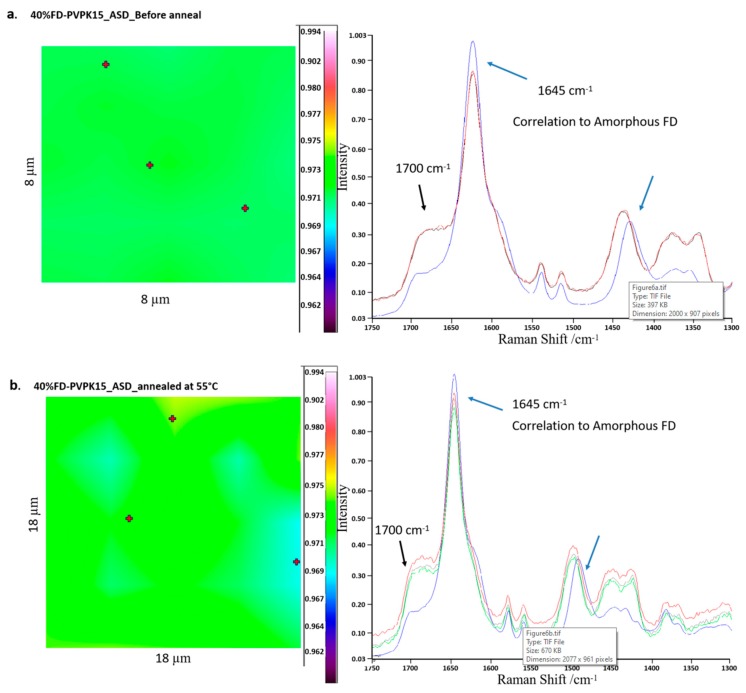

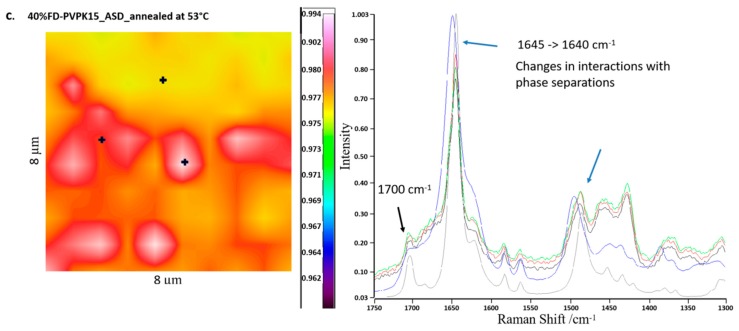
The micro-Raman chemical mapping and corresponding spectra (at selected spots) for FD in the regions between 1750–1300 cm^−1^ for 40%FD-PVPK15 ASDs (**a**) before annealing and after annealing at (**b**) 55 °C and (**c**) 53 °C; the coloured scale bar represents the level of correlation to pure amorphous FD, white/red indicates high correlation and blue/black indicates low correlation.

To further validate the AAPS in tested ASDs, micro-Raman chemical mapping was conducted before and after the annealing (DVS). An example of the micro-Raman chemical mapping on the 40%FD-PVPK15 ASD is shown in Figure 6. The correlation ratio between the sample and pure amorphous FD was ranked as 0–0.99 (BLUE to WHITE with rainbow sequence). A high correlation ratio to aFD will show a RED to WHITE color whilst, a low correlation ratio to aFD will be presented in BLUE. As shown in Figure 5a, the freshly prepared 40% FD-PVPK15 ASD has shown a middle-level correlation with the full aFD at selected Raman shifts between 1750–1300 cm^−1^. Two distinctive Raman scattering peaks at position 1645 and 1700 cm^−1^ can be observed from 40% FD-PVPK15 ASD indicating the amorphous nature of the FD in the ASD, whilst, the position at 1500 cm^−1^ was slightly shifted to higher positions suggesting additional FD-PVPK15 interaction. When 40% FD-PVPK15 ASD was annealed at 55 °C, 90% RH for 6 h, slightly alteration on the correlation of the aFD may be observed (Figure 6b). However, upon close inspections on the spectra, comparable scattering peaks at positions 1645, 1700 and 1500 cm^−1^ to pure aFD were obtained indicating the homogeneous nature of the ASD at the annealing conditions. Following further lowering on the annealing temperature (53 °C, 90% RH, ~6 h), a clear phase separated Raman chemical map was obtained for 40%FD-PVPK15 ASD. Higher correlation areas to pure aFD were colored in RED and/or WHITE (Figure 6c). Through close inspections in the Raman spectra comparison, clear changes can be revealed at all three Raman scattering positions (1645, 1700 and 1500 cm^−1^). The scattering peaks at 1645 and 1500 cm^−1^ from fresh 40% FD-PVPK15 ASD were shifted to 1640 cm^−1^ and 1420 cm^−1^ respectively after annealed at 53 °C, and a sharp peak at 1700 cm^−1^ also appeared. Similar peak positions were also recorded in full crystalline FD (Figure 6c, grey spectrum) albeit in much higher intensity suggesting the potential changes in the molecular interactions have occurred for aFD within the ASD during annealing at 53 °C. However, there was no evidence on the formation of FD crystals (both confirmed by polarized light microscope and high-speed DSC). Similar moisture-induced AAPS in FD-PVPK15 ASD has been reported at conditions of 40 °C, 75% RH and confirmed by confocal Raman mapping [11]. Nevertheless, through the combination of micro-Raman chemical mapping and DSC, we were able to confirm the initiation of the metastable AAPS in FD-PVPK15 ASDs. The results for one phase and AAPS of ASDs after annealing were summarized in Table 2, where the average annealing temperatures were recorded as the binodal boundary for aFD, PVPK15 and water ternary system.

**Figure 7:**
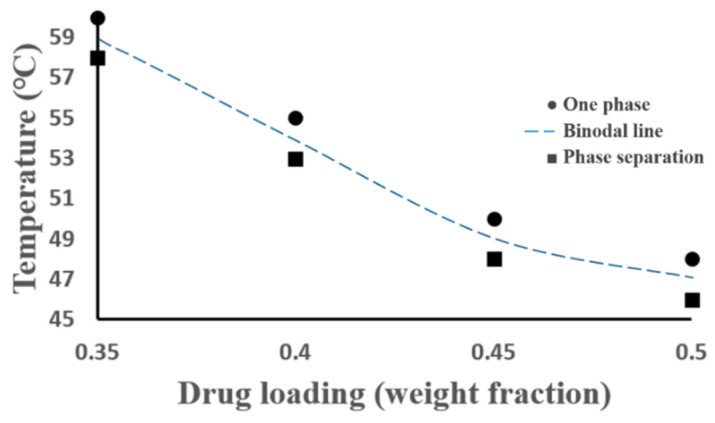
Four experimental points detected for aFD, PVPK15 and water ternary system, the binodal line was the boundary between one phase and AAPS.

In conclusion, four points (triangle symbols) on the binodal line were confirmed by DSC (Figure 5) and micro-Raman chemical mapping, and shown in Figure 7. Circle symbols were verified by the DSC thermogrames as one phase system in Figure 5 (red lines), and square symbols were the conditions of the sample with AAPS observed in Figure 5 (black lines). Triangle symbols were in the average values of the circle and square points, which were further used to model the binodal lines within the ternary system. The corresponding temperatures and drug loadings at AAPS boundary were also summarized in Table 2. Samples annealed in the DVS at 90% RH for six hours were deemed sufficient for the water absorption (Supplementary Materials C), which were also sufficient for detecting the AAPS without substantial crystallization of amorphous FD. Although the phase separation occurs simultaneously for three components in the ternary system, only the aggregations of the amorphous FD from ASD were detected in our study by the high-speed DSC and micro-Raman mapping. The importance of water in a phase separated system might also be investigated by many advanced techniques, such as the transmission electron microscopy (TEM) with selected area electron diffraction (SAED) patterns [58]. The influences of mobile or bonded water on the dynamics of the AAPS, and, subsequently, the crystallization of amorphous drug from the ASD system remain of the highest interests in wide scientific fields.

### 4.5. F–H Interaction Parameters for aFD-PVPK15 at Various Conditions

For binary system, χ ($\Phi_{1},T$) can be experimentally derived at various critical events and their corresponding conditions through the entire temperature and drug loading range. For example, the χ ($\Phi_{1},T$) derived from drug dissolution/melting depression method at high temperature and drug loading range are based on the chemical potential of solid drug and liquid drug in the presence of polymeric carrier Equation (11). At each drug loading of the drug-polymer mixtures, one specific temperature can be identified where the difference between the chemical potential of a solid drug is equal to its liquid form in the mixture (melting event). With this specific temperature and drug loading at this melting event, the χ can be calculated. Similar to this approach, the binodal position between one phase and AAPS provide a critical event where a specific temperature and drug loading can also be used to derive the specific χ. This method has been widely applied for identification of the binodal points in the polymeric mixture and metallic alloy [35,59]. Therefore, combining both methods, a series of χ derived by both dissolution/melting depression and AAPS methods at their corresponding temperatures and drug loadings can be used to obtain the fitting constants of A, B, C and D for Equation (10). We have already reported the values for χ using dissolution/melting depression method for FD-PVPK15, and these values were used in this study [60]. For AAPS approach at low temperature and drug loading range, the values for χ_12_ ($\Phi_{1},T$) at FD-PVPK15 can be directly derived from the χ_12_ ($u_{1},T$) of FD-PVPK15-water ternary system Equations (9) and (10). The results of χ_12_ (FD-PVPK15), χ_13_ (aFD-water) and χ_23_ (PVP K15-water) for water, amorphous FD and polymer PVPK15 ternary system at the binodal boundary are summarized in Table 3. With the construction of the ternary phase diagram, based on the AAPS in aFD-PVPK15 system, it is possible to derive, for the first time, the F–H interaction parameter χ_12_ at low temperatures and drug loadings.

With the obtained values for χ_12_ at various temperatures and drug volume fractions using AAPS method (low temperature and low drug loading) and dissolution/melting depression method (high temperature and high drug loading), it is possible to fit the temperature and composition-dependent χ_12_ for aFD-PVPK15 binary system using Equations (9)–(11). The values of χ_12_ and for FD-PVPK15 across entire experimental temperature and drug loading range were summarized in Table 4.

The fitting constants A, B, C, and D for Equation (10) across entire temperature and drug loading range can be obtained for FD-PVPK15 in this study as:

$$\chi_{12}=1.72+\frac{-852}{T}+5.17\times \Phi_{1}-7.85\times \Phi_{1}^{2}$$

**Figure 8:**
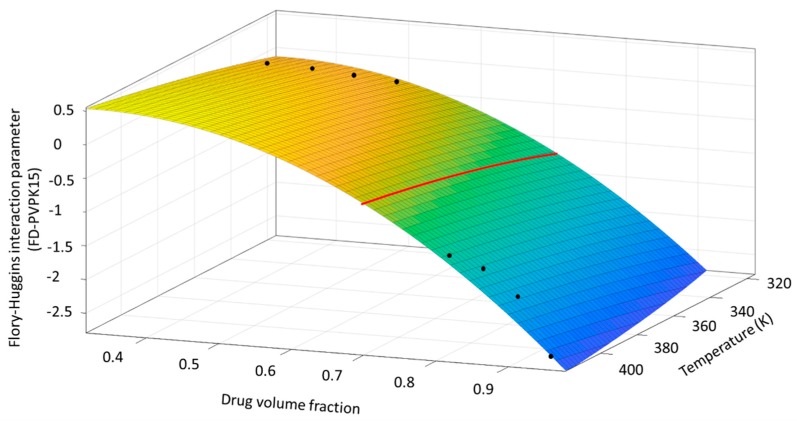
The fitting surface of Equation (10) combined with χ_12_ data from melting depression/dissolution method at high drug loadings high temperatures and AAPS methods at low drug loadings and low temperatures. Black points were χ_12_ data derived from two methods and the red line represent the changes of χ_12_ as a function of temperature when drug loading is fixed.

The goodness of fit for χ_12_ (Φ_1_, T) is R^2^ = 0.98. The fitted surface of Equation (10) was shown in Figure 8, four black points located at high temperature and high drug volume fraction conditions were the values of χ_12_ obtained from our previous work [60]. Other points at lower temperatures and lower drug loadings represent the value of χ_12_ derived from the corresponding condition using AAPS approach.

The temperature and drug loading dependences of χ_12_ obtained from this surface plot (Figure 8) are extremely useful, where the miscibility of the binary system may be directly identified by obtaining the specific F–H interaction parameter at corresponding conditions. For example, as a polynomial equation χ_12_ (Φ_1_, T), when the temperature is constant, e.g., at 46.85 °C (320K), the highest positive value of χ_12_ can be found at drug loading of 33% *v*/*v*. The value of χ_12_ reduces with the drug loading moving away from 33% *v*/*v* (decreases or increases). Furthermore, the temperature-dependence of χ_12_ can also be directly explored via this surface plot. For example, if drug volume fraction sets at a value of 60% (highlight in Figure 8 as the red curve on the surface), a smaller χ_12_ can be obtained at lower temperature indicating a more favorable condition for FD and PVPK15 to form hydrogen bonding [61] It is also very interesting to find that such changes on hydrogen bonding can be reflected through the values of χ_12_ via this surface plot. In a similar system, Grzybowska et al. also suggested that intermolecular hydrogen bonding may break up when the temperature increases (T > T_g_) [22].

As a direct comparison to a recently reported method to fit the constants (high temperatures only), the A, B, C and D fitting constants for FD-PVPK15 were also be identified based on the four points [51]. The A*, B*, C*, and D* obtained for the $\chi_{12}^{*}(\Phi_{1},\;T$) (high temperatures only) were −323, 9.19 × 10^4^, 221, and 122 respectively. This relationship and its fitting surface also illustrated in Figure S3 (Supplementary Materials E). With the χ_12_ ($\Phi_{1}$, T) identified for FD-PVPK15 system across the entire range, as well as the $\chi_{12}^{*}(\Phi_{1},\;T$) identified only using the high temperature values only, it is possible now to obtain the surface plots for Gibbs free energy of mixing for this binary system Equation (13):

(13) $$\frac{∆G}{RT}=\Phi \times \ln\left(\Phi \right)+\frac{\left(1-\Phi \right)}{m}\ln\left(1-\Phi \right)+\chi_{12}\times \Phi \times \left(1-\Phi \right)$$

**Figure 9:**
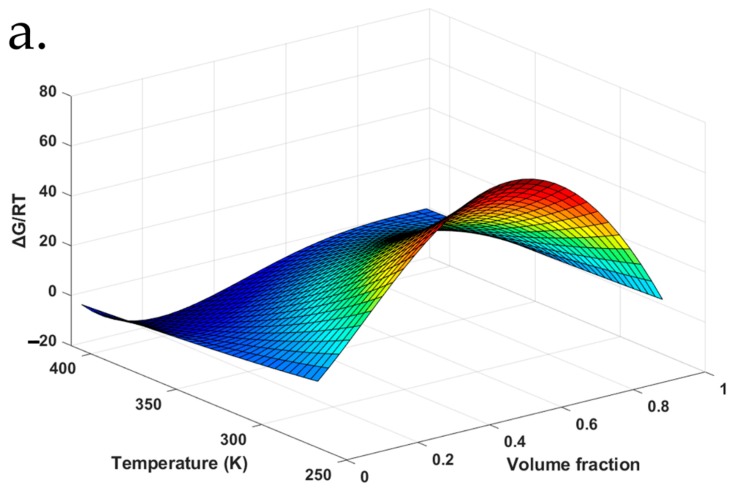

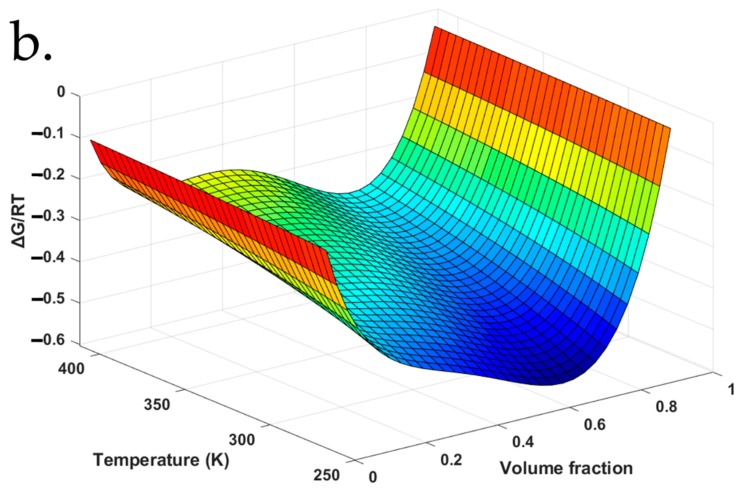
The Gibbs free energy surface for values (**a**) derived from dissolution/melting depression data and (**b**) both dissolution/melting depression and AAPS methods at various temperatures and composition volume fractions.

The Gibbs free energy of mixing for the FD-PVPK15 binary system based on various F–H interaction parameters, $\chi_{12}^{*}(\Phi_{1},\;T$) (high temperature only) and χ_12_ (Φ_1_, T) (entire temperature and drug loading range), were illustrated in Figure 9a,b respectively.

In the current literature, most reported approaches were to obtain single $\chi_{12}$ or $\chi_{12}\left(T\right)$ or $\chi_{12}(\Phi_{1},\;T$) based on the high temperature experimental method [13,14,16,51]. The limitation of these methods will normally lead to an overestimated or underestimated, particularly when the extrapolation of $\chi_{12}$ is normally needed for the prediction of drug-polymer miscibility at lower temperatures (room temperature). Illustrated in Figure 9a with $\chi_{12}^{*}(\Phi_{1},\;T$) derived only from the high temperature method, overall negative ΔG/RT can be observed at high temperature ranges across all drug loadings, whilst the values of ΔG/RT start to shift to positive dramatically when the temperature decreases. A significant increase on the ΔG/RT (positive) as the temperature decreases to 26.85 °C (300K) was observed from the surface plot (Figure 9a) which would suggest the immiscibility between FD and PVPK15 across the entire drug loadings. However, it is clear that a significant discrepancy between the experimental results found in the literature and the F–H based model would suggest the unreliability of this high temperature method. Due to the misinterpretation of the $\chi_{12}^{*}(\Phi_{1},\;T$) and its overall contributions to the ΔG/RT, it has been questioned on whether the F–H theory may not be sufficient for the understanding of miscibility in ASD systems [62]. We proposed in this study to hopefully address this concern by directly deriving the $\chi_{12}(\Phi_{1},\;T$) at both high and low temperature, drug loading ranges where the strong intermolecular interactions may be reflected at these conditions. Through this approach, it will help us to extend the use of F–H theory and highlight the importance of $\chi_{12}(\Phi_{1},\;T$), for the understanding of ASD system. We have utilized $\chi_{12}(\Phi_{1},\;T$) described by the values of $\chi_{12}$ from both high and low temperature and drug loading ranges to construct the ΔG/RT surface plot (Figure 9b). In comparison to Figure 9a, the most noticeable difference was the negative values of Gibbs free energy of mixing for aFD-PVPK15 system, where good miscibility can be found at the entire range of temperatures and drug loading. The good miscibility for FD-PVPK15 system was also widely reported with various drug loadings (30~74% *w*/*w*) at temperatures from 0 °C to 150 °C in many other research articles [63]. With this combined approach, the intermolecular interactions between the aFD and PVPK15 at entire temperature and drug loading range were indeed better reflected in the $\chi_{12}(\Phi_{1},\;T$).

### 4.6. FD-PVPK15 Binary Phase Diagram

**Figure 10:**
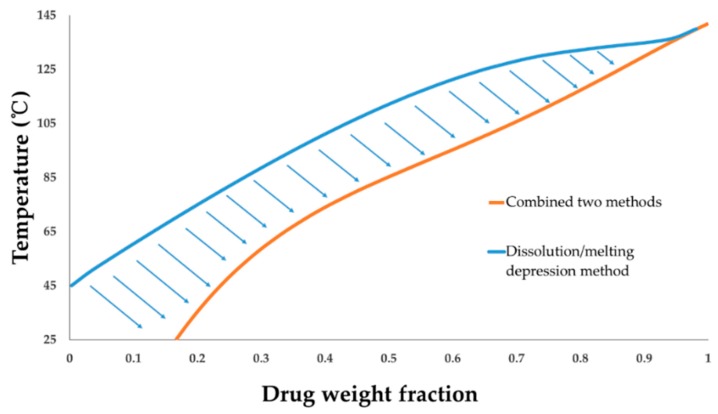
The phase diagram constructed using $\chi_{12}^{*}\left(\Phi_{1},\;T\right)$ from dissolution/melting depression method (blue solid line) and χ_12_ (Φ_1_, T) from the entire temperature and drug loading range (orange solid line).

With the new set of aFD-PVPK15 interaction parameters, $\chi_{12}(\Phi_{1},\;T$), obtained at both high and low temperatures and drug loadings, it is possible for the first time to update the drug-polymer temperature-composition phase diagram with greater certainty. Compared with the F–H interaction parameter only obtained from the dissolution/melting depression method ($\chi_{12}^{*}(\Phi_{1},\;T$)), the newly constructed phase diagram based on the F–H interaction parameter, $\chi_{12}(\Phi_{1},\;T$), was shown in Figure 10. The solid orange line is the FD solubility curve in polymer PVPK15 modelled using $\chi_{12}(\Phi_{1},\;T$), and the blue solid line is the FD solubility curve in PVPK15 modelled based on the $\chi_{12}^{*}\left(\Phi_{1},\;T\right)$ (high temperature only). It is interesting to see that the blue curve extrapolated by the $\chi_{12}^{*}(\Phi_{1},\;T$) obtained using high temperature method suggested a significant underestimation on the drug solubility of FD in PVPK15, especially at low temperature and drug loadings. The overestimated Gibbs free energy of mixing for aFD-PVPK15 system based on the $\chi_{12}^{*}\left(\Phi_{1},\;T\right)$ at low temperature conditions (Figure 9a) might lead to the underestimated miscibility and solubility of this drug-polymer binary system. As we discussed above, a stronger hydrogen bonding between drug and polymer at low temperature (T < T_g_) can significantly enhance the tendency of mixing of the ASD which should be carefully considered through the model. With the orange solid curve (Figure 10), the $\chi_{12}(\Phi_{1},\;T$) based on data from entire conditions provide a more realistic representation of FD-PVPK15 ASD system using the combination of AAPS and dissolution/melting depression approach. Particularly, a solubility of 16% *w*/*w* FD is predicted in PVPK15 system at 25 °C, which is more close to the experimental results often reported in the literature [63,64].

More importantly, the Flory–Huggins interaction parameter $\chi_{12}(\Phi_{1},\;T$) across the entire temperature and composition range was successfully obtained through carefully designed experiments and a more complex modelling. With the $\chi_{12}(\Phi_{1},\;T$) derived from this combinational approach, the understanding of the aFD-PVPK15 binary system can be further enhanced, e.g., the modelling for kinetics of phase behaviors in none-equilibrium ASDs where the time associated to the demixing may be predicted [57]. Thus, a comprehensive predictive tool encompassing both thermodynamic and kinetic aspects of physical stability may be subsequently obtained in the future.

## 5. Conclusions

The establishment of an informative framework is imperative for the understanding of drug-polymer ASD system, particularly at pharmaceutical relevant temperatures and drug compositions. A comprehensive framework can be utilized as the foundation for QbD and implemented in the design and optimization of stable ASD formulations. The current methods for obtaining the thermodynamic phase diagram of a drug-polymer binary system can be achieved by measuring the drug dissolution/melting depression data or the T_g_ of ASD via demixing. However, the limitations associated with both methods are the fact that, at high temperatures, the drug and polymer systems were treated as ideal solutions and variations of types and strengths of drug-polymer interactions were not considered. In this work, we have presented for the first time an experimental approach to obtain the drug loading and temperature-dependent drug-polymer F–H interaction parameters at low temperatures via amorphous-amorphous phase separation (AAPS) mechanism. This occurrence of AAPS was further expedited using water as the third component at carefully selected experimental conditions. Through the detection of binodal points for FD, PVPK15, and water ternary system at the low temperatures, the corresponding FD-PVPK15 interaction parameters were also successfully derived. The F–H interaction parameter $\chi_{12}(\Phi_{1},\;T$) as functions of temperature and drug loading was obtained from the entire range. With these values for FD-PVPK15 interaction parameters,${\;\chi }_{12}(\Phi_{1},\;T$), we now can improve the understanding of FD-PVPK15 miscibility at various temperatures and drug loadings, and obtain the drug-polymer temperature-composition phase diagram with better representations at pharmaceutical relevant conditions. For future work, with the understanding of $\chi_{12}(\Phi_{1},\;T$), it is now possible to simulate the kinetics of phase separation and predict the physical stability of a non-equilibrium amorphous solid dispersion, and subsequently establish a reliable QbD framework for ASD formulation development.

## Figures and Tables

**Table 1:** The calculated F–H interaction parameters and their fitted coefficients of Equations (B3) and (B4) (Supplementary Materials B) for aFD-water and PVPK15-water system at 30 °C, 40 °C, 50 °C, and 60 °C, data were presented with average ± standard deviations for F–H interaction parameters (*n* = 3).

Χ	Temperature of Dynamic Vapor Absorption Experiment (°C)							
	30		40		50		60	
χ_13_ (aFD-water)	5.68 ± 0.05		3.73 ± 0.07		2.16 ± 0.37		2.06 ± 0.31	
χ_23_ (PVPK15-water)	0.497 ± 0.01		0.404 ± 0.03		0.169 ± 0.05		−0.116 ± 0.02	
	B	A	a_0_	a_1_	b_0_	b_1_	c_0_	c_1_
aFD-water	1.27 × 10^4^	−36.5	/	/	/	/	/	/
PVPK15-water	/	/	−68.5	2.16 × 10^4^	185	−5.79 × 10^4^	−120	3.76 × 10^4^

Where A, B, a_0_, a_1_, b_0_, b_1_, c_0_, c_1_ were coefficients in Equations (B3) and (B4) which shown in Supplementary Materials B, experiments were repeated three times.

**Table 2:** The summary of AAPS boundary at drug loading 35%, 40%, 45% and 50%.

Number of Glass Transition	Temperature of one Phase to AAPS (°C) at Different Drug Loadings			
	35%	40%	45%	50%
One glass transition	60	55	50	48
Two glass transitions	58	53	48	46

**Table 3:** Data of F−H interaction parameter χ and volume fraction of drug, water, and polymer ternary system at the AAPS boundaries conditions (*n* = 3).

Temper-ature (°C)	Volume Fraction (%)			F–H interaction Parameters		
	FD	PVPK15	Water	χ_12_ * (aFD-PVPK15)	χ_13_ (aFD-Water)	χ_23_ (PVPK15-Water)
47	44.5 ± 0.2	45.2 ± 0.4	10.2 ± 0.4	−0.284	3.11	0.651
49	39.9 ± 1.0	49.5 ± 1.0	10.7 ± 1.0	−0.219	2.86	0.662
54	34.8 ± 0.5	53.0 ± 0.5	12.3 ± 0.5	−0.0823	2.26	0.693
59	29.9 ± 0.8	56.4 ± 0.8	13.7 ± 0.8	0.0293	1.68	0.724

* χ_12_ was calculated by solving OBJ function Equation (8) using known parameters listed in Table 3. Experiments were repeated three times, and standard deviations were derived from the water content measured by DVS.

**Table 4:** Data of F–H interaction parameter χ_12_ (Φ_1_, T) for FD-PVPK15 obtained via both AAPS (low temperatures) and dissolution/melting depression (high temperatures) methods. (*n* = 3).

Temperature (°C)	Volume Fraction (%)		χ_12_ Derived from Two Different Methods
	FD	PVPK15	
141.5 ± 0.2	94.9	5.08	−2.66 *
140.5 ± 0.1	89.9	10.1	−1.84 *
139.2 ± 0.4	84.8	15.2	−1.48 *
137.3 ± 0.3	79.8	20.2	−1.40 *
59	34.6 ± 0.8	65.4 ± 0.8	0.0293 ^§^
54	39.6 ± 0.5	60.4 ± 0.5	−0.0823 ^§^
49	44.6 ± 1.0	55.4 ± 1.0	−0.219 ^§^
47	49.6 ± 0.4	50.4 ± 0.4	−0.284 ^§^

* Data were obtained from our previous work, dissolution/melting depression approach [60]; ^§^ data were obtained from AAPS approach.

## Supplementary Materials

The following are available online at https://www.mdpi.com/1999-4923/11/8/420/s1, A: MATLAB Scripts for Solving the Objective Function of Binodal Curve in Ternary System. B: Flory-Huggins interaction parameters of drug-water (χ_13_) and PVPK15-water (χ_23_). C: Metastable state for ASD samples annealed at 90% RH and various temperatures at binodal line. D: The crystallization of FD from AAPS systems after prolonged period of annealing at 90% RH. E. The Flory-Huggins interaction parameter χ_12_ derived only from AAPS or dissolution/melting depression method.
