# Supplementary material for: The Investigation of Flory–Huggins Interaction Parameters for Amorphous Solid Dispersion Across the Entire Temperature and Composition Range
Source: Pharmaceutics. 2019 Aug 19;11(8):420. doi: 10.3390/pharmaceutics11080420 (PMC6722828; doi:10.3390/pharmaceutics11080420)
Supplement: Supplementary file 1 [file pharmaceutics-11-00420-s001.pdf]

pharmaceutics

Supplementary Materials: The Investigation of Flory-Huggins Interaction Parameters for Amorphous Solid Dispersion Across the Entire Temperature and Composition Range

Yiwei Tian [1,*], Kaijie Qian [1], Esther Jacobs [1], Esther Amstad [2], David S. Jones [1], Lorenzo Stella [3,4] and Gavin P. Andrews [1]

A. MATLAB Scripts for Solving the Objective Function of Binodal Curve in Ternary System

Script A1

```
%%This is a test script to show the usage of the function find_ch12


clear

close all

clc


% Indices

% 1 = drug

% 2 = polymer

% 3 = water


vsite = 1.8016e-5/1; % for water, assuming density = 1. g/cm^3. Unit: m^3/mol

v1 = 16.678*vsite; % for drug. Unit: m^3/mol

v2 = 440.917*vsite; % for polymer. Unit: m^3/mol

v3 = vsite; % for water. Unit: m^3/mol


% Flory-Huggins parameters (experimental)

chi31 = 3.7984; % water-drug

chi23 = 0.303; % polymer-water


% The model parameters are collected into a vector
```

```
pars = [vsite, v1, v2, v3, chi31, chi23];


% drug and water volume fractions (experimental)

phi0 = [0.47024, 0.4777];


% initial guess

chi0 = -1;


% find the value of chi12 solving the equivlent variational problem

chi12 = find_chi12(phi0, chi0, pars);


disp(['  chi12 = ', num2str(chi12)])
```

Script A2

```
%%find_chi12 finds the value of chi12 for which the point phi0 lies on the

%binodal line.

%

%   chi12 = find_chi12(phi0, chi0, pars)

%

%   phi0 = [phi0(1), phi0(2)] is the 2-dim vector containing the drug and

%   water volume fractions of the mixture (experimental).

%   By definition, the polymer fraction is: 1-phi0(1)-phi0(2).

%   phi0 is supposed to lie on the binodal line.

%

%   chi0 is the initial guess of the Flory-Huggins parameter (dimensionless)

%   for drug-polymer interaction.

%

%   pars = [vsite, v1, v2, v3, chi31, chi23] is a vector containing the

%   relevant model parameters. vsite (unit: m^3/mol) is the molar volume of
```

```
%   the lattice site of the underlining lattice model. It is kept here for
%   back compatibility. v1, v2, v3 (unit: m^3/mol) are the molar volumes of
%   the three components (1=drug, 2=polymer, 3=water). chi31 and ch23 are
%   the value of the (dimensionless) Flory-Huggins parameters for the
%   drug-water and polymer-water interaction (experimental).
%
%   The function returns the value of chi12 found solving the equivalent
%   variational problem.


function chi12 = find_chi12(phi0, chi0, pars)
```

Constants

1 = drug 2 = polymer 3 = water

```
vsite = pars(1);
v1 = pars(2);
v2 = pars(3);
v3 = pars(4);


chi31=pars(5);
chi23=pars(6);


%T = 300*Rg; % temperature. Unit. J
```

symbolic functions

```
syms phi1 phi2 chi12


% This is the Gibbs free energy of mixing. Unit: dimensionless
dG_mix = ((phi1/v1)*log(phi1)+(phi2/v2)*log(phi2)...
    +((1-phi1-phi2)/v3)*log(1-phi1-phi2)+(chi12/vsite)*phi1*phi2...
```

```
    +(chi23/vsite)*phi2*(1-phi1-phi2)...
    +(chi31/vsite)*(1-phi1-phi2)*phi1)*vsite;

% This is the chemical potential of mixing (drug). Unit: dimensionless
mu1_mix = dG_mix+diff(dG_mix, phi1, 1)*(1-phi1)...
    -diff(dG_mix, phi2, 1)*phi2;

% This is the chemical potential of mixing (polymer). Unit: dimensionless
mu2_mix = dG_mix+diff(dG_mix, phi2, 1)*(1-phi2)...
    -diff(dG_mix, phi1, 1)*phi1;

% This is the chemical potential of mixing (water). Unit: dimensionless
mu3_mix = dG_mix-diff(dG_mix, phi1, 1)*phi1...
    -diff(dG_mix, phi2, 1)*phi2;

% The next two lines are meant to check the internal consistency
%dG_mix_check = phi1*mu1_mix+phi2*mu2_mix+(1-phi1-phi2)*mu3_mix;
%simplify(dG_mix-dG_mix_check)
```

Function handles

```
% Those are functions of: (phi1, phi2, chi12) in this given order
%f_dG_mix = matlabFunction(dG_mix, 'Vars', [phi1, phi2, chi12]);

f_mu1_mix = matlabFunction(mu1_mix, 'Vars', [phi1, phi2, chi12]);
f_mu2_mix = matlabFunction(mu2_mix, 'Vars', [phi1, phi2, chi12]);
f_mu3_mix = matlabFunction(mu3_mix, 'Vars', [phi1, phi2, chi12]);

d2_mu_1 = @(chi12, phi1x, phi2x, phi1y, phi2y) ...
    (f_mu1_mix(phi1x, phi2x, chi12)-f_mu1_mix(phi1y, phi2y, chi12)).^2;
```

```
d2_mu_2 = @(chi12, phi1x, phi2x, phi1y, phi2y) ...
    (f_mu2_mix(phi1x, phi2x, chi12)-f_mu2_mix(phi1y, phi2y, chi12)).^2;

d2_mu_3 = @(chi12, phi1x, phi2x, phi1y, phi2y) ...
    (f_mu3_mix(phi1x, phi2x, chi12)-f_mu3_mix(phi1y, phi2y, chi12)).^2;
```

Cost (or objective) function

```
Fcost = @(chi12, phi11, phi21) 0.5*(...
    d2_mu_1(chi12, phi11, phi21, phi0(1), phi0(2))...
    +d2_mu_2(chi12, phi11, phi21, phi0(1), phi0(2))...
    +d2_mu_3(chi12, phi11, phi21, phi0(1), phi0(2)));
```

Optimisation

```
% initial values
phi11=0.4;
phi21=0.4;

X0 = [chi0, phi11, phi21];

func = @(x) Fcost(x(1), x(2), x(3));

nonlcon = @(x) penalty(phi0, [x(2), x(3)]);

options = optimset('Display', 'Iter', 'TolX', 1.e-7, 'TolFun', 1.e-14);

[Xsol, Fval] = fmincon(func, X0, [], [], [], [], [], [], nonlcon, options);

disp(['  chi12 = ', num2str(Xsol(1))])
```

```
disp(['  phi0 (ref) = ', mat2str([phi0(1), phi0(2), 1-phi0(1)-phi0(2)], 5)])

disp(['  phi1 (fit) = ', mat2str([Xsol(2), Xsol(3), 1-Xsol(2)-Xsol(3)], 5)])

disp(['  phi0 and phi1 must be different. If they are too close, please change the value of
sigma used by the penalty function.'])

disp('  Chem. potentials @ phi0 (ref) = ')
disp(mat2str([f_mu1_mix(phi0(1), phi0(2), Xsol(1)),...
f_mu2_mix(phi0(1), phi0(2), Xsol(1)),...
f_mu3_mix(phi0(1), phi0(2), Xsol(1))], 5))

disp('  Chem. potentials @ phi1 (fit) = ')
disp(mat2str([f_mu1_mix(Xsol(2), Xsol(3), Xsol(1)),...
f_mu2_mix(Xsol(2), Xsol(3), Xsol(1)),...
f_mu3_mix(Xsol(2), Xsol(3), Xsol(1))], 5))

disp(['  The value chemical potentials for phi0 and phi1 should be equal within a few
decimal places to have a reliable solution.'])

disp(['  Residual = ', num2str(Fval)])

disp(['  The residual must be small to ensure a reliable solution.'])

[c, ceq] = penalty(phi0, [Xsol(2), Xsol(3)]);

disp(['  Penalty (c) = ', num2str(c)])
```

```
disp(['  The penalty must be smaller than zero.'])


chi12 = Xsol(1);

end
```

PENALTY

This function makes sure that phi1 is different from phi0. This is done using an inequality condition (see below)

```
function [c, ceq] = penalty(phi0, phi1)
% This is the minimal distance of phi1 and phi0.
% WARNING: This value may need to be adjusted
sigma = 0.05;

% This is an inequality condition:
% norm(phi1-phi0) > sigma
c = sigma-norm(phi1-phi0);

ceq = []; % Not used, but required by the fmincon syntax
end
```

Published with MATLAB® R2018b

B. Flory-Huggins interaction parameters of drug-water (χ_{13}) and PVPK15-water (χ_{23})

The relationships between FD-water and PVPK15-water interaction parameters as a function of water vapor pressure may be obtained using dynamic vapor sorption experiments (DVS). The relationship between water activity a_w and water-excipient interaction parameters may be described as Equations. (B1) and (B2): [1]

$$\ln a_w = \ln \phi + (1-\phi)[(1-1/M) + \chi(1-\phi)] \quad \text{(B1)}$$

$$\chi(a_w) = \chi(0) + B_1 \times a_w + B_2 \times a_w^2 \quad \text{(B2)}$$

ϕ is the volume fraction of absorbed water in the sample, M is the number of segments of the polymer molecules denoted. The Equation (B1) demonstrated the water activity can be described by the drug loading ϕ and interaction parameter χ. Meanwhile, the Equation (B2) describes the interaction parameter χ as a function of a_w, coefficients B_1 and B_2 are constant at a certain temperature, where $\chi(0)$ is the F-H interaction parameter for drug-water system. It is noted that the amorphous

drug should be used to measure the drug-water interaction parameter (χ_{13}) as we proposed to investigate the amorphous-amorphous phase separation in the ternary system. Since the concentration independent parameter for FD-water, χ_{13}, was utilized in this study, the relationship between the temperature and χ_{13} may be simplified as:[2]

$$\chi_{13} = A + \frac{B}{T} \tag{B3}$$

where *A* is the entropic related term and *B* is the enthalpy related term. Polynomial relationship was used to describe the both concentration and temperature dependent parameters for PVPK15-water system $\chi_{23}(u_2,T)$:[3]

$$\chi_{23}(u_2, T) = \left(a_0 + \frac{a_1}{T}\right) + \left(b_0 + \frac{b_1}{T}\right) \times u_2 + \left(c_0 + \frac{c_1}{T}\right) \times u_2{}^2 \tag{B4}$$

where a_0, a_1, b_0, b_1, c_0, and c_1 are the polynomial coefficients, $u_2 = \varphi_2 / (\varphi_2 + \varphi_3)$.

C. Metastable state for ASD samples annealed at 90% RH and various temperatures at binodal line

In Figure S1, the examples of dynamic water absorption curves were presented for FD-PVPK15 ASD samples at drug loading (a) 35%; (b) 40%; (c) 45% and (d) 50% *w/w* annealed at AAPS boundary conditions of 59 ℃ (a), 54 ℃ (b), 49 ℃ (c) and 47 ℃ (d) up to 8 h. It is clear that the steady state for water uptakes from these ASDs was reached before 6 h when the correct conditions were chosen. It was also verified that these systems were remained on the binodal line (co-existing line between one phase and AAPS) and crystal-free during the entire 8 h annealing under 90% relative humidity.

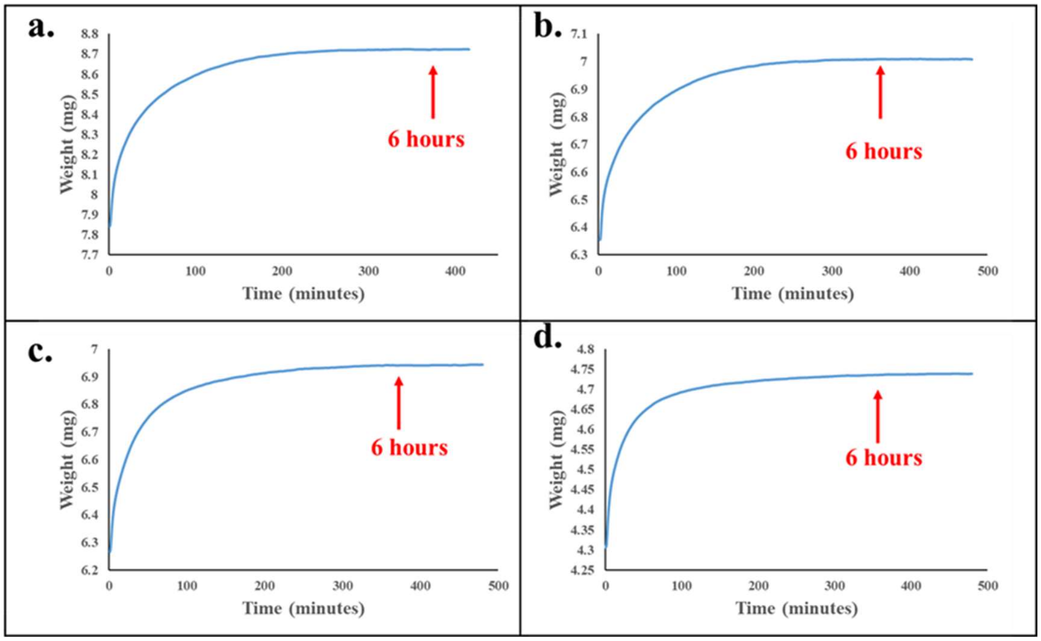

Figure S1. Dynamic water absorption for ASD samples **(a).** 35% w/w; **(b).** 40% w/w; **(c).** 45% w/w; **(d).** 50% w/w annealed at the AAPS boundary conditions **(a).** 59℃; **(b).** 54℃; **(c).** 49℃; **(d)**. 47℃ for 8 h, all samples were annealed at 90% RH.

D. The crystallization of FD from AAPS systems after prolonged period of annealing at 90% RH

The crystallization of amorphous FD after phase separation can be verified via the DVS process. For example, for FD-PVPK15 ASD containing 50% *w*/*w* FD annealed within the binodal region (45 °C, 90% RH) for six h (Figure S2. a), it was clear that amorphous-amorphous phase separated system with crystallized FD can be obtained after extending this annealing experiments time for 9 h (Figure S2. b) and 15 h (Figure S2. c). There were two main phenomena commonly observed during an extended annealing under high humidity condition: (1) as the annealing time increases, the value for first Tg started to decrease (drug-rich phase, Figure S2. a-b). (2) The amorphous drug from drug-rich domains started to crystallize after 8 under AAPS conditions. The level of phase separation was increased as the annealing time increases further (6 to 9, 15 h) until the crystallization happened, where the amorphous drug composition started to decrease, shifting to crystalline drug.

a-6 h

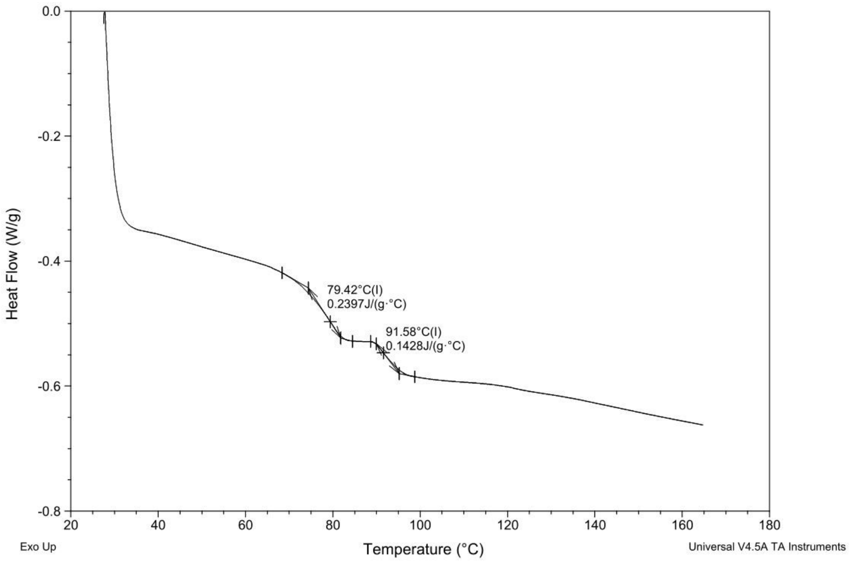

b-9 h

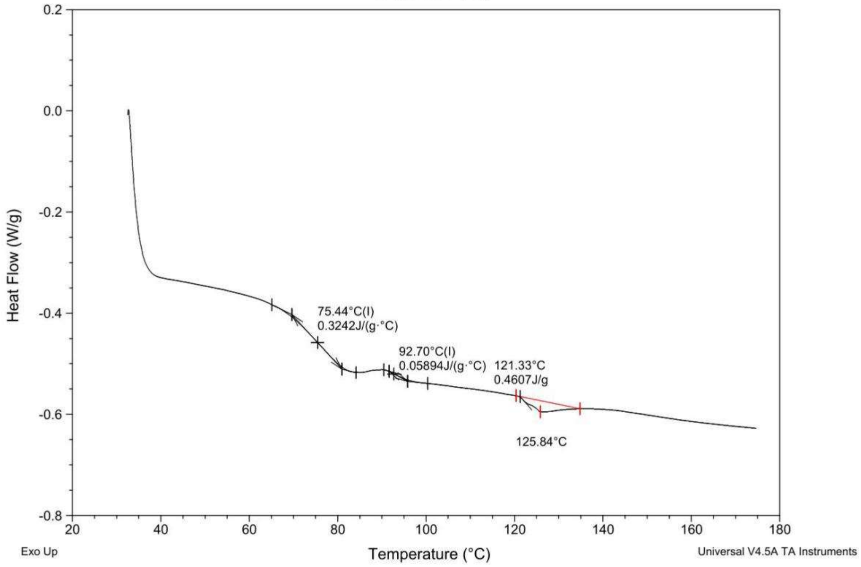

c-15 h

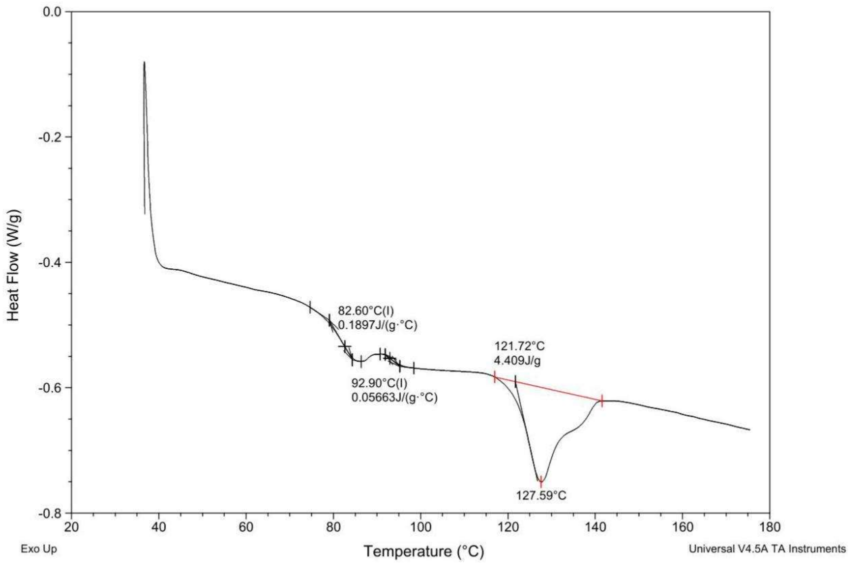

Figure S2. DSC thermogram of the co-existence of amorphous-amorphous phase separation and crystallized FD after annealed at 46℃, 90% RH for 6 h **(a)**, 9 h **(b)**, and 15 h **(c)**.

E. The Flory-Huggins interaction parameter χ_{12} derived only from AAPS or dissolution/melting depression method

The fitting surfaces of temperature and drug volume fraction dependences F-H interaction parameter χ_{12} derived from only AAPS method at low temperature low drug loading or dissolution/melting depression method at high temperature high drug loading range were illustrated in Figure S3. a and b respectively. Four fitting coefficients for Equation. (10) (main text) derived from two methods individually can be obtained and the relationship may also describe as:

$$\chi_{12}^{\S} = 9.01 + \frac{-3255}{T} + 3.77 \times \Phi_1 - 4.04 \times \Phi_1^2$$

$$\chi_{12}^{*} = -323 + \frac{91910}{T} + 220 \times \Phi_1 - 122 \times \Phi_1^2$$

where $\chi_{12}^{\S}$ and χ_{12}^{*} were the values of FD-PVPK15 F-H interaction parameter derived only from AAPS or the dissolution/melting depression approach. A significant difference for fitting coefficients and its fitting surfaces were observed from two methods at corresponding conditions. Compared with the values of χ_{12} derived from combined methods, there is no single approach can reliably reflect both temperature and drug volume fraction dependences F-H interaction parameter χ_{12} across the entire condition. Therefore, the combination of the AAPS method at low temperature low drug loading and dissolution/melting depression approach at high temperature and drug loading was utilized in this study.

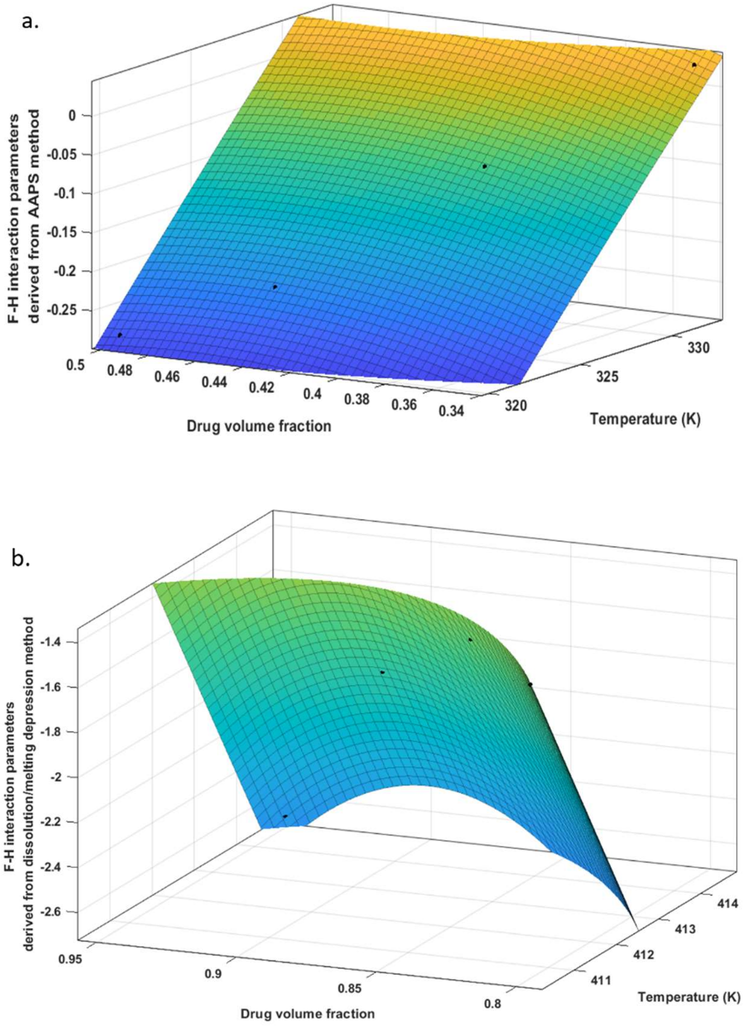

Figure 3. The fitting surface of χ_{12} data only from the **(a).** AAPS at low temperatures low drug loadings or **(b).** melting depression/dissolution method at high temperatures high drug loadings. Black points were $\chi_{12}^{\S}$ and χ_{12}^{*} data derived from two methods.

Reference

1. Beck, M.I.; Tomka, I. Effects of the degree of substitution in ethyl cellulose on the clustering of sorbed water. *J. Macromol. Sci. Part B* **2007**, *36*, 19–39.
2. Tian, Y.; Booth, J.; Meehan, E.; Jones, D.S.; Li, S.; Andrews, G.P. Construction of drug-polymer thermodynamic phase diagrams using flory-huggins interaction theory: Identifying the relevance of temperature and drug weight fraction to phase separation within solid dispersions. *Mol. Pharm.* **2013**, *10*, 236–248.
3. Schuld, N.; Wolf, B.A. Solvent quality as reflected in concentration- and temperature-dependent Flory-Huggins interaction parameters. *J. Polym. Sci. Part B Polym. Phys.* **2001**, *39*, 651–662.
